# Phenotypic error threshold; additivity and epistasis in RNA evolution

**DOI:** 10.1186/1471-2148-5-9

**Published:** 2005-02-03

**Authors:** Nobuto Takeuchi, Petrus H Poorthuis, Paulien Hogeweg

**Affiliations:** 1 Theoretical Biology/Bioinformatics Group, Utrecht University, Padualaan 8, 3584 CH Utrecht, The Netherlands

## Abstract

**Background:**

The error threshold puts a limit on the amount of information maintainable in Darwinian evolution. The error threshold was first formulated in terms of genotypes. However, if a genotype-phenotype map involves redundancy ("mutational neutrality"), the error threshold should be formulated in terms of phenotypes since there is no unique fittest genotype. A previous study formulated the error threshold in terms of phenotypes, and their results showed that a rather low degree of mutational neutrality can increase the error threshold unlimitedly.

**Results:**

We obtain an analytical formulation of the phenotypic error threshold by considering the "additive assumption", in which base substitutions do not influence each other (no epistasis). Our formulation shows that an increase of the error threshold due to mutational neutrality is limited. Computer simulations of RNA evolution are conducted to verify our formulation, and the results show a good agreement between the analytical prediction and the simulations. The comparison with the previous formulation illustrates that it is important for the prediction of the error threshold to consider that the number of base substitutions per replication is rather large near the error threshold. To examine the additive assumption, a detailed analysis of additivity and epistasis in RNA folding of a particular sequence is performed. The results show a high degree of epistasis in RNA folding; furthermore, the analysis also elucidates the reason of the success of the additive assumption.

**Conclusions:**

We conclude that an increase of the error threshold by mutational neutrality is limited, and that the additive assumption achieves a good prediction of the error threshold in spite of a high degree of epistasis in RNA folding because the average number of base substitutions of sequences retaining the phenotype per replication is sufficiently small to avoid of the effect of epistasis.

## Background

The error threshold is a limit on the permissible mutation rate for which "survival of the fittest" holds in Darwinian evolution [1]. The error threshold can be seen as a limit on the amount of information maintainable in evolutionary systems (information threshold) since an increase in sequence length results in an increase in error rate. The information threshold leads to a paradox in prebiotic evolution [2]. Suppose that to increase the maintainable amount of information, an evolving system must acquire a more complex molecular mechanism to reduce the mutation rate. However, to have such a complex molecular mechanism the system must maintain a longer sequence in the first place. Thus, the system will encounter a barrier in the evolution of complexity (cf. [3]).

The error threshold was first formulated in terms of genotypes. However if some changes in genotype do not alter the phenotype or the fitness (mutational neutrality), there is no unique genotype which can be stably maintained. Instead, the survival of a phenotype should be considered, and thus the error threshold should be formulated in terms of phenotypes [4].

In this paper, first we formulate the phenotypic error threshold analytically by employing the additive assumption, in which base substitutions do not influence each other. Under the additive assumption, we obtain the probability that a replication does not alter the phenotype (neutral replication) as a function of the number of base substitutions and the fraction of "neutral substitutions". Our results show a qualitative difference from the previous formulation [5]. Second, we analyze epistasis in RNA folding of a particular RNA sequence to examine the additive assumption.

## Results and discussion

### Phenotypic error threshold

#### Analytical formulation

The quasispecies equation describes (prebiotic) replicator dynamics in well-mixed systems [1]. We transform the equation in two ways: (1) describing the abundance of phenotypes instead of that of genotypes by denoting the population of genotypes which share the same phenotype by one variable (see [5] for mathematical details); (2) distinguishing only two classes of phenotypes, the focal phenotype (denoted by *x*) and the others called mutants (denoted by *y*). The population size is assumed to be large enough to express the abundance of the phenotypes by normalized concentration. The population dynamics of the phenotypes is described as

*dx*/*dt *= *σQx *+ *σ*Λ (1 - *Q*)*x *- *Dx *- Φ*x*,

*dy*/*dt *= *y *+ *σ *(1 - Λ)(1 - *Q*)*x *- *Dy *- Φ*y *    (1)

where *σ *(> 1) is the replication rate of *x *(that of *y *is normalized to 1); *Q *is the replication accuracy of *x*. Λ is the fraction of neutral mutants of *x*. *D *is the degradation rate (or the death rate) assumed to be uniform over the phenotypes. Φ = (*σ *- *D*)*x *+ (1 - *D*)*y *is the excess production (or the mean fitness). The terms - Φ*x *and - Φ*y *induce a selection pressure. We neglect back mutation from *y *to *x *(this simplification will be discussed in the next section). From Eq. 1, we obtain the survival condition of *x *as *Q *+ Λ (1 - *Q*) >*σ*^-1^, for which the stationary value of *x *is larger than zero. From this inequality, we will deduce the phenotypic error threshold, i.e., the maximum error rate of replication for which *x *can be stably maintained in the system.

We distinguish two classes of single base substitutions: neutral and deleterious substitutions. The class of a substitution is determined by the effect of the substitution on the phenotype when there are no other substitutions in the genotype: A substitution which retains the phenotype is a neutral substitution; otherwise it is a deleterious substitution. A beneficial substitution is not considered since the focus of the study is on the maintenance of *x*. [A replicator is thought of as a polymer in our study. We refer to a monomer as a base, having RNA in mind. The formulation of the phenotypic error threshold itself is independent of this terminology.]

To calculate the effective replication accuracy *Q*_*e *_= *Q *+ Λ (1 - *Q*), we assume that a mutant is neutral *iff *there is no deleterious substitution: The effect of a substitution on the phenotype is independent of the other substitutions; i.e., no epistasis is assumed (the additive assumption). Let *λ *denote the fraction of neutral substitutions in all possible single substitutions, and let *d *denote the number of substitutions per replication. Then, the probability that *d *substitutions are all neutral substitutions (thus neutral replication) is approximated by *λ*^*d *^by assuming that the number of neutral substitutions in *d *substitutions follows the binomial distribution (the binomial approximation). This approximation is valid if the probability of correct replication per base (denoted by *q*) is sufficiently large so that *d *is small. Denoting the sequence length of replicators by *N*, the effective replication accuracy (*Q*_*e*_) is obtained as



by assuming that *q *and *λ *are uniform among the genotypes in *x*, that *q *is invariable over sequence positions, and that *N *is the same among the populations. [A similar formula was obtained in [6] as *Q*_*e *_= (*q *+ *v*(1 - *q*))^*N*^, where *v *is a parameter to be tuned to match the formula to the observed value of *Q*_*e*_. Therefore *v *in [6] implicitly involves both the additive effect and epistasis.]

The minimum *q *for which *x *can survive is derived from *Q *+ Λ(1 - *Q*) >*σ*^-1 ^and Eq. 2 as

*q*_min _= (*σ*^-1/*N *^- *λ*)/(1 - *λ*).     (3)

**Figure 1:**
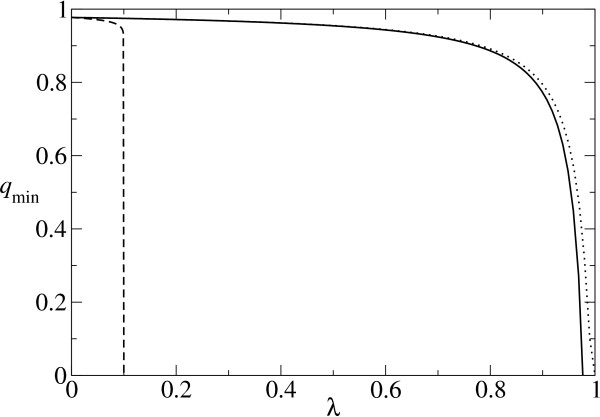
**Error threshold **The minimum permissible replication accuracy per base (*q*_min_) is plotted against *λ *for three different ways of the calculation. The solid line is obtained from the additive assumption and the binomial approximation (Eq. 3). The binomial approximation is threatened at a high error rate. To examine this, the error threshold is calculated without the binomial approximation (but with the additive assumption) in the extreme example, where the binomial approximation deviates most (see the text). Let *N*_*δ *_be the sum of the sequence length of the parts where all single mutations are deleterious. Then *q*_mim _is calculated as . The dotted line represents the so calculated error threshold in this extreme example. The *x*-axis for the dotted line (i.e., *λ*) is calculated as (*N *- *N*_*δ*_)/*N*. The dashed line is obtained from the formulation of Reidys *et al. *[5] (Eq. 5). In all cases, *N *= 100 and *σ *= 10. (The same values of *N *and of *σ *as those used in [5] are chosen for a comparison purpose.)

The phenotypic error threshold is 1 - *q*_min_. As seen in Fig. 1 (the solid line), the increase in the error threshold is limited for almost all values of *λ*. This is because if *q *decreases, the number of substitutions per replication (*d*) increases; hence the probability of neutral replication (*λ*^*d*^) decreases (cf. [5] and Eq. 5). At a large *λ *(= *σ*^-1/*N*^), there is a singularity such that *q*_min _becomes zero. However, this singularity is not plausible in two ways: (1) Such a large *λ *is not realistic (see below); (2) *q *at the singularity is so small that the binomial approximation is threatened. We studied the validity of the binomial approximation, and found that the inaccuracy in the binomial approximation is largest if in some positions of the sequence all possible single substitutions are neutral, but in the rest of the positions all possible single substitutions are deleterious; i.e., the distributions of neutral and deleterious substitutions over the sequence positions are completely separated. By taking this extreme example, *q*_min _is calculated with the additive assumption but without the binomial approximation. As Fig. 1 (the dotted line) shows, for a wide range of *λ *the binomial approximation is valid. *q*_min _is underestimated by the binomial approximation only around the singularity (*λ *> 0.8), and thus the singularity is actually located at higher *λ*, which makes the singularity even less plausible. We conclude that the increase in the error threshold due to mutational neutrality is limited.

From Eq. 3, we obtain the information threshold, i.e., the maximum permissible sequence length as

*N*_max _= ln(*σ*^-1^)/ln(*q *+ (1 - *q*)*λ*).     (4)

**Figure 2:**
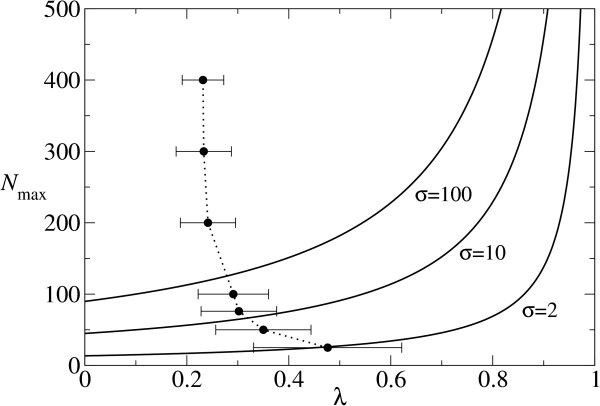
**Information threshold **The solid lines represent the maximum maintainable sequence length (*N*_max_) plotted against *λ*, where *N*_max _is calculated by using Eq. 4 where *σ *= 2, 10 and 100 (as indicated within the figure) and *q *= 0.95. The value of *q *was chosen to be plausible for ribozyme polymerization [14]. These solid lines show the dependence of *N*_max _on *λ*; on the other hand, the dependence of *λ *on *N *(the length of sequence) is examined by calculating the average *λ *in RNA folding for various values of *N*. The filled circles represent so obtained *λ *values as a function of *N*. In obtaining *λ*, a neutral substitution is defined as a single base substitution which does not alter the secondary structure of a focal sequence. For each sequence length, a hundred randomly generated RNA sequences are examined. In RNA folding program [7], the default parameters are used (in the all occasions in the study).

As Fig. 2 (the solid lines) shows, the increase in the information threshold is limited for plausible values of *λ*. (*N*_max _reaches infinity only when *λ *increases to one.) However, this result does not mean that a longer sequence in fact can have a larger *λ*, and thus it can be maintained. We studied the relationship between *λ *and the sequence length by utilizing RNA folding, which is a well-studied prototype of genotype-phenotype map, where the genotype is the primary structure of an RNA sequence and the phenotype is the minimum free energy secondary structure of the RNA sequence. We utilized Vienna RNA package [7] to fold RNA (The default parameters are used in the all occasions in the study). The average *λ *for different sequence length was obtained through comparing the secondary structure of randomly created RNA sequences with that of all possible mutants with only one substitution. A substitution which retains the original secondary structure is considered as neutral; otherwise deleterious. As Fig. 2 (the filled circles) shows, the average *λ *is a decreasing function of the sequence length. This relationship further limits an increase of information threshold due to mutational neutrality.

#### Comparison between the analytical prediction and computer simulations

We compare our analytical prediction with computer simulations. Our computer program simulates the evolution of RNA replicators in a well-mixed flow reactor (e.g. see [8]). In the simulations, each RNA sequence replicates and/or is diluted (be taken out from the reactor) with a certain probability in every time step. RNA folding is utilized again as a genotype-phenotype map (computed by [7]): The fitness of an RNA sequence depends on the secondary structure (i.e., the phenotype) of the RNA sequence. The fittest phenotype is set to the secondary structure of a yeast tRNAphe (the clover leaf structure, *N *= 76). RNA sequences which have the fittest phenotype replicate with the probability 0.01 per time step; all the other RNA sequences (mutants) replicate with the probability 0.001 per time step (thus *σ *= 10). The replication introduces mutations with a certain probability. Back mutations are not allowed to occur – the effect of back mutations is negligible if the sequence length is large enough [9] (this was confirmed by the simulations which were the same as the above except for allowing back mutations [data not shown]). The dilution probability (Φ) is calculated as the average probability of replication divided by the target population size. The target population size is set to 10000. All the simulations start with 10000 yeast tRNAphe sequences. The degradation of sequences is ignored (*D *= 0).

The results of the computer simulations showed that the "representative *λ*" (defined in Methods section – Non-uniform distribution of *λ*) of the fittest sequences increased from 0.307 to ca. 0.40 for the examined values of the error rate (data not shown). (This is also true for the population average of *λ *[op. cit.].) The value of the representative *λ *fluctuates over the time (st. dev. = 0.01 at 1 - *q *= 0.0475).

**Figure 3:**
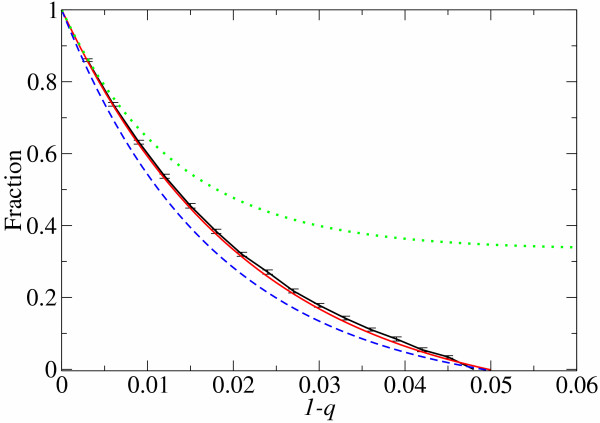
**Comparison of population structure between analytical predictions and computer simulations **The fraction of the fittest phenotype population is plotted against the error rate per base (1 - *q*). The black line (the solid line with error bars) is obtained from computer simulations. The red line (the solid line without error bars) is calculated with the additive assumption from Eq. 1 and Eq. 2 with *σ *= 10 and *N *= 76 as in the simulations. *λ *is set to be 0.4 which is the time averaged representative *λ *value observed in the simulations after the population evolves (see the Methods section – Non-uniform distribution of *λ *– for the definition of the representative *λ*). The green line (the dotted line) is obtained from the formulation of Reidys *et al. *[5] with the same parameters as the above. The blue line (the dashed line) is calculated with the four *λ *approximation by using *λ *values reported in [5] (*λ *= 0.2489 on average) and *σ *= 10 and *N *= 76 as in our simulations.

The equilibrium fraction of the fittest sequences of the computer simulations is compared to that of the analytical prediction over the different error rate in Fig. 3. The analytical prediction is calculated under the additive assumption from Eq. 1 and Eq. 2 by using the time averaged representative *λ *observed in the simulations after the evolution (*λ *= 0.4). As Fig. 3 shows, the calculation (the [red] solid line without error bars) closely predicts the result obtained from the computer simulations (the [black] solid line with error bars). The predicted error threshold (0.05) is slightly higher than that observed (between 0.045 and 0.048) probably due to the assumption of infinite population in Eq. 1 (see [3,10]).

#### Comparison with a previous formulation

Reidys *et al. *[5] derived the phenotypic error threshold as



from *Q*_*e *_= *Q *+ *λ *(1 - *Q*). This equation shows an unlimited increase in the error threshold for *λ *≥ *σ*^-1 ^(see the dashed line in Fig. 1 and the [green] dotted line in Fig. 3). However, *Q*_*e *_= *Q *+ *λ *(1 - *Q*) is valid only if either (1) a neutral set is uniformly distributed over the genotype space [a neutral set is a set of genotypes where all genotypes map to the same phenotype], or (2) *q *is so large that most mutants have *d *= 1. The uniform distribution of neutral sets in the genotype space is not applicable in RNA folding as shown later. The latter possibility is discussed next.

**Figure 4:**
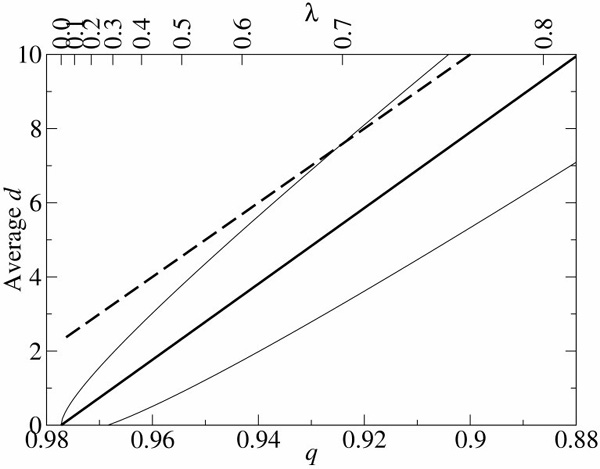
**Number of substitutions per replication in mutants **The *y*-axis is the number of base substitutions (*d*) per replication (or per neutral replication) at the error threshold. The thick solid line represents the average *d *per neutral replication (i.e., the average *d *of the sequences which retain the master phenotype per replication): *d *= *Np*/(*q*_mim _+ *p*) where *p *= *λ *(1 - *q*_min_) and *λ *= (*σ*^-1/*N *^- *q*_min_)/(1 - *q*_min_). The thin solid line represents the standard deviation of it, i.e., ± (*q*_mim _+ *p*) *Np *. The dashed line represents the average *d *per replication, which is *N*(1 - *q*_min_). *N *is 100 and *σ *is 10. The lines are plotted against *q*_min _(the lower *x*-axis), and the corresponding *λ *is shown in the upper *x*-axis.

Studies of replicator dynamics on a neutral network often consider a very large value of *q *so that most mutants have *d *= 1 (e.g., [11]). [A neutral network is a neutral set, or its subset, where every genotype is connected to at least one genotype of the set by one or two base substitutions.] However, if the error rate (1 - *q*) is close to the error threshold, mutants can have on average *d *> 1 even if *λ *= 0, for which the error threshold is at the lowest error rate (see the dashed line in Fig. 4). The average *d *of the neutral sequences (i.e., the exact copies and the neutral mutants) per replication (this will be later called the average *d *per neutral replication) is lower than the average *d *per replication. However at the error threshold, even the average *d *per neutral replication is larger than one for *λ *> 0.32 (see Fig. 4, the solid lines). Above consideration asserts that the error threshold will be substantially overestimated if one considers only a single mutation.

Reidys *et al. *[5] obtained an extension of Eq. 5, the so called "four *λ *approximation". This extension divides a sequence in four sub-sequences in order to take into account the fact that the fraction of neutral substitutions varies over the sequence position. This extension still overestimates *Q*_*e *_though less so than Eq. 5 because the approximation now permits four substitutions per replication as a side effect of the subdivision. Note that this extension makes a fairly good prediction on the error threshold (see the [blue] dashed line Fig. 3) because the use of a small non-evolved *λ *value coincidentally cancels out the overestimation.

In conclusion, it is crucial for the calculation of the error threshold to consider that the number of substitutions per replication is large near the error threshold.

### Epistasis in RNA folding

The rather impressive success of the additive assumption is counter-intuitive in view of RNA folding, in which many interactions occur between bases. In the next part of the paper, we study a particular RNA sequence, namely the yeast tRNAphe (which comprises the initial population of the previously described RNA evolution simulations), in terms of additivity and epistasis. The objective of this study is to understand how the additive assumption achieves a good prediction in spite of a high degree of nonlinearity in RNA folding [12,13].

We compare the secondary structure of randomly sampled mutants to that of the tRNAphe. Similar to the previous section, a mutant is neutral if its secondary structure is the same as that of the tRNAphe; otherwise, it is deleterious. To evaluate the deviation from the additive assumption, we categorize mutants into four classes as shown in Table 1. Negative epistasis refers to a mutant which is predicted to be neutral under the additive assumption, but turns out to be deleterious due to the interaction of the base substitutions. Positive epistasis refers to the reverse case.

**Table 1:** Definition of positive and negative epistasis.

	mutants are	
	neutral	deleterious
*δ *= 0	additive neutral	negative epistasis
*δ *> 0	positive epistasis	additive deleterious

*δ *is the number of deleterious base substitutions.

**Figure 5:**
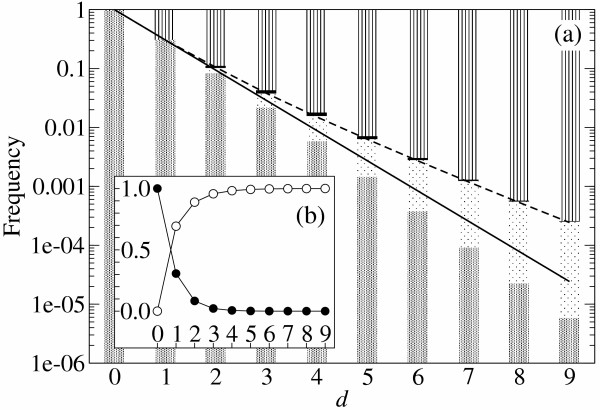
**Additivity and epistasis in RNA folding **The frequency of mutant classes is plotted against the number of base substitutions (*d*). **(a) **Log. plot. The patterns in the bars indicate the mutant classes: (from bottom) mesh, additive neutral; dots, positive epistasis; black, negative epistasis; stripes, additive deleterious (see Table 1 for the definition). The data were generated by RNA folding (by using [7]) with a *S. cerevisiae *tRNAphe sequence as a reference sequence: GCGGAUUUACCUCAGUUGGGAGAGGGCCAGACUGAACAUCUGGAGGUCCGGCGCGCGAUACGCCGAAUUCGCACCA (each non-RNA is converted to RNA). We examined all possible mutants at *d *= 1, 2 and the subsets of mutants for other *d *values (2<*d*<10, the portion of examined mutants is respectively, 10, 1, 0.1, 0.01, 0.3 × 10^-3^, 0.1 × 10^-3^, 0.4 × 10^-5^%). These observations in RNA folding are compared with the following two analytical predictions. The solid line is the probability of neutral replication estimated under the additive assumption (*λ*^*d*^, *λ *= 0.307). The dashed line is the probability of neutral replication estimated with epistasis ((*d*), see Methods section – Probabilistic approach). **(b) **Linear plot. Symbols: ● the frequency of the neutral mutants (additive neutral and positive epistasis); ○ the frequency of the deleterious mutants (additive deleterious and negative epistasis).

As Fig. 5 shows, the additive assumption underestimates the degree of mutational neutrality. The same conclusion was drawn differently in [13], where the additive neutral mutant is defined as a neutral mutant which lies in the same neutral network as that of the original sequence. Our results show that positive epistasis occurs more frequently than negative epistasis in total. What actually happens is as follows. If mutants with *δ *> 0 are only considered, positive epistasis occurs very rarely compared to additive deleterious case: No more than 0.5% of the mutants are neutral at *d *= 5 if they carry at least one deleterious substitution. If mutants with *δ *= 0 are only considered, negative epistasis is rather frequent relative to additive neutral case: As much as 35% of the mutants are deleterious at *d *= 5 even if they carry only neutral substitutions. However, replication with *δ *> 0 occurs far more frequently than replication with *δ *= 0: As much as 99.7% of the replication contains at least one deleterious substitution at *d *= 5 and *λ *= 0.307. Therefore, the relative frequency of epistasis is flipped around. Consequently, the additive assumption underestimates the degree of mutational neutrality. (Note that in Fig. 3 the additive assumption predicts the fraction of the fittest sequences always slightly smaller than that of the computer simulations.)

**Figure 6:**
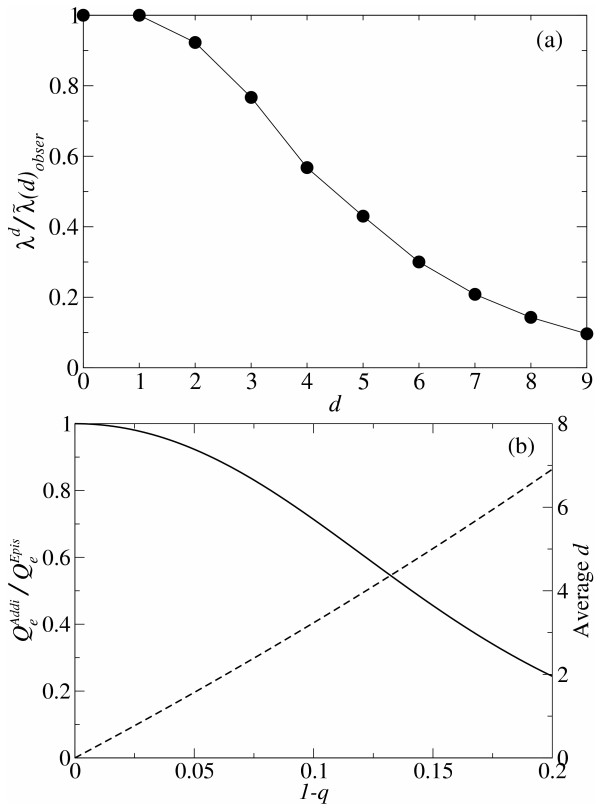
**Comparison between additivity and epistasis in RNA folding (a) **The *relative *probability of neutral replication under the additive assumption (*λ*^*d*^) is plotted against the number of base substitutions (*d*), where the probability of neutral replication with epistasis (i.e. the fraction of neutral mutants observed in the yeast tRNAphe folding) is set to be 1 for each *d *as a reference. It can be seen that the effect of epistasis on the probability of neutral replication becomes larger as the number of substitution (*d*) increases. The same data as that of Fig. 5 are used. **(b) **The solid line (the left *y*-axis) represents the *relative *effective replication accuracy (*Q*_*e*_) under the additive assumption plotted against the error rate (1 - *q*), where *Q*_*e *_calculated with epistasis is set to be 1 as reference (see Methods section – Probabilistic approach – for details). It can be seen that the effect of epistasis on *Q*_*e *_increases as the error rate (1 - *q*) increases. The shape of the curve is in a similar manner as that of the curve in Fig 6a. Although the *x*-axis of Fig 6b is different from that of Fig 6a, one can relate the two graphs via the average *d *per neutral replication, with which the different *x*-axes can be transformed to each other. The average *d *per neutral replication is represented by the dashed line (the right *y*-axis plotted against 1 - *q*). Its value is calculated under the additive assumption as *d *= *Np*/(*q *+ *p*) where *p *= *λ *(1 - *q*), *λ *= 0.4 and *N *= 76.

The effect of epistasis is already noticeable when *d *> 2 as seen in the comparison between the probability of neutral replication under the additive assumption (*λ*^*d*^) and that observed in RNA folding (see Fig. 6a). Since the average *d *per replication is more than 3 close to the error threshold in our simulations, Fig. 6a may seem to suggest that the additive assumption would substantially underestimate the effective replication accuracy (*Q*_*e*_) near the error threshold.

We calculate the effective replication accuracy (*Q*_*e*_) including the effect of epistasis in order to compare it with *Q*_*e *_calculated under the additive assumption. The first trial was to include a "trivial" epistasis in base paired regions (helices) as a part of the additive effect (see Methods section – Trivial epistasis). However, the analysis showed that epistasis occurs mainly in a "non-trivial" way (data not shown), and thus it is not sufficient for our sake to include a trivial epistasis. We next took a probabilistic approach to calculate *Q*_*e *_with epistasis (see Methods section – Probabilistic approach). The results of this method agree with the observation (see the dashed line in Fig. 5).

We compare *Q*_*e *_calculated under the additive assumption to that calculated with epistasis as shown in Fig. 6b (the solid line). As the comparison shows, the additive assumption indeed underestimates *Q*_*e*_; however, the underestimation becomes prominent only if the error rate is higher than the error threshold (1 - *q*_min _= 0.05). As Fig. 6b (the dashed line) shows, the average *d *per neutral replication is ca. 1.5 at the error threshold, which is much smaller than the average *d *per replication (ca. 3.8). This means that the main contribution to *Q*_*e *_under the additive assumption is from the mutants of *d *= 1 or 2 at the error threshold. According to Fig. 6a the additive assumption is a good approximation at *d *= 1 or 2. Therefore, the additive assumption accurately estimates *Q*_*e *_and thus the error threshold. When the average *d *per neutral replication reaches 3, the additive assumption substantially underestimates *Q*_*e *_(see Fig. 6b), which is consistent with Fig. 6a. [However, note that this analysis does not imply that the average number of substitutions per replication is safely assumed to be near one. On the contrary, in our calculation it was more than one – actually 3.8 – at the error threshold.]

In the above examination of the additive assumption, there are two points which must be examined further: (1) The analysis of epistasis was performed on a yeast tRNAphe, which comprises the initial population of the RNA evolution simulations, but the results may differ if the analysis is done for a sequence which appears later in the RNA evolution simulations. Thus, we performed the same analysis to a sequence which was chosen from the population of the fittest sequences after the evolution in the simulations (at the 20000th time step). The results, however, did not change our conclusion (data not shown). (2) If the length of sequences is larger, the average *d *per neutral replication may increase, and thus the additive assumption may break down before the error threshold. However, it turns out from the analytical calculation that the average *d *per neutral replication at the error threshold decreases as *N *increases when *λ *is invariant (cf. the caption of Fig. 6b). Furthermore, *λ *decrease as *N *increases (see the filled circles in Fig. 2). Therefore, if the sequence length is larger, the average *d *per neutral replication will be actually smaller. We also conducted computer simulations of RNA evolution with a longer sequence length (200 bases). The results showed that the average *d *per neutral replication (calculated under the additive assumption) at the error threshold was indeed smaller (ca. 1.2 substitutions with *λ *≈ 0.35) than in the previous case of the shorter sequence length (ca. 1.5 substitutions with *λ *≈ 0.4), and the additive assumption still predicts the results of the simulations closely (data not shown).

## Conclusions

• The phenotypic error threshold was formulated under the additive assumption. The formulation asserted that mutational neutrality increases the error threshold but the increase is limited.

• The importance of considering multiple substitutions per replication at the error threshold was illustrated.

• The comparison with the computer simulations and the analysis of epistasis showed that the additive assumption correctly estimates the effective replication accuracy (*Q*_*e*_) and thus the error threshold.

• The reason why the additive assumption achieves a good prediction of the error threshold in spite of a high degree of (non-trivial) epistasis in RNA folding is that the average number of substitutions per *neutral *replication is small enough to avoid of the effect of epistasis.

## Methods

### Non-uniform distribution of *λ*

If *λ *is not uniform over the genotypes sharing the same phenotype, the effective replication accuracy (*Q*_*e*_) depends on the distribution of the genotypes in the population. In this case, *Q*_*e *_is calculated under the additive assumption as



where *X*_*I *_denotes the population of the focal phenotype (*I*), and *x*_*i *_(resp. *λ*_*i*_) is the population (resp. the fraction of neutral substitutions) of the genotype *i*. The set *S*_*I *_denotes the set of genotypes which have the phenotype *I*. If *x*_*i *_and *λ*_*i *_are known, the representative *λ *of the phenotype can be calculated from the following equation as



The difference between the representative *λ *and the population average of *λ *was very small in the computer simulations. (The population average was always slightly smaller [ca. 99%] than the representative *λ *unless the distribution of *λ *in the fittest population is completely homogeneous [data not shown].)

### Calculation of *Q*_*e *_with epistasis

#### Trivial epistasis in RNA folding

It is trivial that epistasis occurs between bases which make a pair (hydrogen bond) in the reference secondary structure. Our first trial to include epistasis in the calculation of *Q*_*e *_was to include this epistasis as a part of the additive effects of mutations as described in [5]. In this approach, the reference sequence is subdivided into non-paired regions and paired regions; paired regions are treated as strings of base pairs (one pair of bases is considered as one character); a substitution of a base pair is considered as an elementary step of mutations in paired regions. Following this procedure, the epistasis occurring between bases in a pair is now treated as an additive effect. [For example, two mutations – GC→GG and GC→CC – occurring in paired base must be deleterious because the bases can not make a pair any more. Given that the combined mutation – GC→CG – is neutral, it will be a case of positive epistasis in the previous procedure. However, in the new procedure it will be a case of additive neutral because the combined mutation is treated as one substitution of a base pair.] We categorized the mutants into the previously defined four groups of mutants (i.e. additive neutral, additive deleterious, positive epistasis and negative epistasis) using the same data as that of Fig. 5. However, the result did not differ much from that shown in Fig. 5 (data not shown). We conclude that epistasis occurs mainly in a non-trivial way, and thus this approach is not effective for our purpose.

#### Probabilistic approach

Since (non-trivial) epistasis makes it difficult to predict what happens to the phenotype given a specific change in genotype, we take the following probabilistic approach: We assume that a mutant is neutral with a certain probability (denoted by *μ*(*v*, *δ*)), which depends on the number of neutral base substitutions (denoted by *v*) and on that of deleterious base substitutions (denoted by *δ*). Then, the probability of neutral replication is obtained (by using the binomial approximation) as



where *d *= *v *+ *δ*. *Q*_*e *_is thereupon derived as



**Figure 7:**
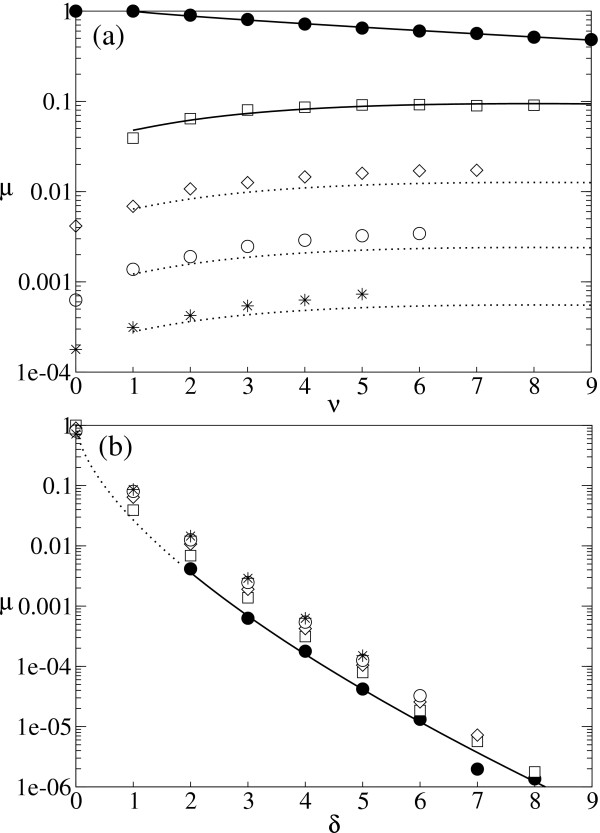
**Probabilistic approach in calculating the effective replication accuracy with epistasis (a) **The probability that a mutant is neutral with *v *neutral substitutions and *δ *deleterious substitutions (i.e., *μ*(*v*, *δ*)) is plotted against the number of neutral substitutions (*v*). Symbols: ● *δ *= 0; □ *δ *= 1; ◇ *δ *= 2; ○ *δ *= 3; * *δ *= 4. The plots were obtained from the same data set as that of Fig. 5. The solid lines represent the results of curve fitting. We used Eq. 10 (*v*>0, *δ *= 0) to the *δ *= 0 data set, and Eq. 10 (*v*>0, *δ*>0) to the *δ *= 1 data set. The second fitting was done after we obtained *α *and *ε*_*n *_from the first fitting, and *ε*_*d *_and *η *from the fitting in Fig. 7b. The dotted lines are the estimation made with the obtained parameters (listed below). **(b) ***μ*(*v*, *δ*) plotted against the number of deleterious substitutions (*δ*). Symbols: ● *v *= 0; □ *v *= 1; ◇ *v *= 2; ○ *v *= 3; * *v *= 4. The solid part of the line represents the curve fitting; the dotted part is an exception, i.e., *μ*(0, 1) = 0. We used Eq. 10 (*v *= 0, *δ*>0) toward the *v *= 0 data set in the fitting. All the fitting was done after transforming both the equations and the data sets to logarithmic scale to reduce the biased importance of the points in small *d*. The obtained parameters are as follows: *ε*_*n *_= 0.1190, *α *= 0.8483, *ε*_*nd *_= 2.418, *β *= 2.333, *γ *= 3.996, *ε*_*d *_= 0.02697 and *η *= 0.6380.



We measured *μ*(*v*, *δ*) in the tRNAphe folding as shown in Fig. 7ab. When *δ *= 0 and *v *> 0, *μ *declines a little slower than exponentially as *v *increases due to negative epistasis (Fig. 7a). When *v *= 0 and *δ *> 1, *μ *is not zero due to positive epistasis, and *μ *decreases slower than exponentially as *δ *increases (Fig. 7b). When *v *> 0 and *δ *> 0, *μ*(*v*, *δ*) increases, saturates, and finally decreases as *v *increases (Fig. 7a): neutral substitutions can compensate deleterious substitutions. We express the above observations as follows:

where *ε*_*n*_, *ε*_*d *_and *ε*_*nd *_are the epistatic parameters of the interactions among neutral substitutions, among deleterious substitutions, and between neutral and deleterious substitutions, respectively. Note that in the additive assumption, all epistatic parameters are zero. *α *and *η *represent non-exponential decay. To express the compensation by neutral substitutions, we arbitrarily used a saturation function *βv*/(*γ *+ *v*) where *β *and *γ *are parameters. To obtain the parameters, we fitted Eq. 10 to the data in Fig. 7ab (the solid lines) as explained in the caption. As shown in Fig. 7a (the dotted lines), the theoretical estimation turns out to be a slight underestimation. (*d*) was calculated from the above obtained parameters, and the calculated values match the observed ones (see the dashed line in Fig. 5).

## Authors' contributions

NT contributed to the entire part of the study. PHP contributed to the computer programing of the RNA evolution simulation. PH contributed to the conceptual development of the study and the manuscript preparation as the supervisor.
